# Don’t Get Discouraged

**Published:** 1879-03

**Authors:** 


For Hall's Journal.

### DON'T GET DISCOURAGED.

"I am almost discouraged," is the language of many an invalid reader; and for you are these lines penned; you may have been ill for weary months, but look for better days, and don't get discouraged. You may have a family to care for, and may be far from them, but if you would be restored to them in blooming health, don't get discouraged.

You may have difficulties to encounter, and obstacles to overcome, and be far too, from your physician, with no one to give you a look, or a word of encouragement, but notwithstanding all this, don't get discouraged, but stop for a moment, and think, then contrast your condition with that of many others, and see how many less cares you have than they; but after all, they don't get discouraged.

May be you think, that there is no prospect of your ever recovering—but indulge the hope that there is, and don't get discouraged. Perhaps your physician has often told you, that "if you don't maintain a cheerful frame of mind, it will retard your recovery." Take his advice, and don't get discouraged. But it may be your life is fast passing away. "For all flesh is as grass, and all the glory of man as the flower of grass; the grass withereth, and the flower thereof falleth away: but the word of the Lord endureth forever." You have the promise, if you labor for your Master here, of receiving an immortal crown, as a reward for your labors. Be patient then, and you may soon be admitted into the new Jerusalem, where no discouragements will ever afflict you.

MAHALA

